# Objective Cognition Impairment Comparisons between Sexual Minority and Non‐Sexual Minority Older Adults

**DOI:** 10.1002/alz70857_106269

**Published:** 2025-12-25

**Authors:** Carolina L. Costa, Carlos E.E. Araujo Menendez, Rachel Membreno, Ariana M Stickel

**Affiliations:** ^1^ San Diego State University, San Diego, CA, USA; ^2^ SDSU/UC San Diego Joint Doctoral Program in Clinical Psychology, San Diego, CA, USA

## Abstract

**Background:**

Sexual minorities (SM) refer to individuals who identify as having non‐hetero sexual identities. Compared to non‐SM, SM groups face challenges in mental health, accessing healthcare, and discrimination, heightening their risk for adverse health outcomes, possibly including cognitive impairment. Limited research has examined whether there are disparities in cognitive impairment between SM and non‐SM individuals using objective measures, and findings have been inconsistent. We aimed to investigate the risk of objective cognitive impairment between SM and non‐SM individuals.

**Method:**

This study included 1794 participants (SM = 85, non‐SM = 1709) ages 64‐95 years (mean = 73.62 years, SD = 6.40) from the 2016 Harmonized Cognitive Assessment Protocol (HCAP), a sub‐study of the Health and Retirement Study (HRS). The SM category included those who identified their sexual identity as lesbian, gay, bisexual, or other, and the non‐SM category included those who identified as non‐SM. Outcomes included cognitive impairment status (yes, no) in visuospatial, orientation, memory, executive function, and language and fluency domains as established through factor analysis models by Jones and colleagues (2023). Logistic regression models were used to assess the association between cognitive impairment and SM status, adjusting for age, marital status, education, race/ethnicity, and Hispanic heritage

**Result:**

Compared to non‐SM participants, SM individuals were more likely to identify as Hispanic (38.8% vs 8.6%) and to report having no educational degree (36.5% vs 11.8%). Adjusting for covariates, there was a trending association of SM status on orientation impairment such that SM people were more likely to have orientation impairment than non‐SM individuals (OR = 2.01, 95% CI [.99, 4.09]). No other cognitive impairment differences by SM group were detected.

**Conclusion:**

Overall, levels of cognitive impairment were largely similar between SM and non‐SM groups. However, the SM group was trending toward a higher risk for orientation impairment. This domain specificity may reflect acute changes to cognition rather than long‐standing cognitive impairment. Further research is needed to determine 1) if these findings remain in larger, more diverse samples and 2) the possible biopsychosocial mechanisms underlying these findings.

